# Development of an Eco-Friendly Smartphone-Assisted Nystagmus Recording System for Recording Vertigo Attacks Anytime, Anywhere: Pilot App Development Study

**DOI:** 10.2196/73811

**Published:** 2025-06-18

**Authors:** Yumi Dobashi, Masao Noda, Tatsuaki Kuroda, Noriaki Miyata, Makoto Ito, Reiko Tsunoda, Hiroaki Fushiki

**Affiliations:** 1 Department of Otolaryngology, Mejiro University Ear Institute Clinic, 320 Ukiya, Iwatsuki-ku, Saitama-shi, Saitama, 339-8501, Japan, 81 48-797-3341; 2 Department of Otolaryngology and Head and Neck Surgery, Jichi Medical University, Shimotsuke, Japan; 3 Kuroda ENT Clinic, Yatsushiro-shi, Kumamoto, Japan

**Keywords:** dizziness, telemedicine, vertigo, “iCapNYS” system, mobile health, smartphone

## Abstract

**Background:**

The widespread adoption of smartphones and tablet devices, along with advancements in data communication technology, has resulted in a paradigm shift in the treatment of dizziness. External factors, such as the spread of COVID-19, have accelerated this transformation in recent years. We have been pursuing telemedicine and web-based medical care to treat dizziness and have developed different products and services necessary for each treatment process stage. Several patients face difficulties in accessing medical facilities during severe vertigo episodes. Furthermore, clinical findings, such as nystagmus or other symptoms, may be absent when symptoms subside by the time of their appointment.

**Objective:**

This study aimed to develop a smartphone app for capturing eye movements and head positions during vertigo attacks, enabling recordings anywhere, even at home or work.

**Methods:**

We developed an app named “iCapNYS” that uses the iPhone’s front camera and gyro sensor to record eye movements and head positions. The app incorporates features designed to encourage spontaneous eye movements, minimizing nystagmus suppression caused by fixation. Additionally, we designed lightweight, recyclable cardboard goggles to securely hold the smartphone and block visual stimuli from the surrounding environment, optimizing the recording conditions.

**Results:**

The “iCapNYS” system successfully captured subtle peripheral vestibular nystagmus in a patient with vertigo. The recorded nystagmus characteristics are comparable to those obtained using traditional infrared CCD (charge-coupled device) cameras.

**Conclusions:**

This app is an effective tool for treating vertigo and is easy for older adults to use, as it can be recorded with only 3 taps. We expect that the introduction of this nystagmus-monitoring system will improve vertigo treatment quality, promote medical collaboration, and provide patients with peace of mind in their care.

## Introduction

Vertigo and dizziness are symptoms of various disorders, including peripheral vestibular, central, cardiovascular, and psychogenic disorders. The 1-year prevalence and incidence of vertigo in adults are 4.9%, and 1.4%, respectively [1]. These conditions, which lead to episodic vertigo attacks, reduce the patients’ quality of life by causing nausea and vomiting, accompanied by headaches or an increased risk of falls [2]. Among diseases, benign paroxysmal positional vertigo (BPPV) is one of the most frequent causes of episodic vertigo; its 1-year prevalence and incidence were 1.6% and 0.6%, respectively [3]. The 1-year prevalence of vestibular migraine is estimated to be 1% to 2.7% [4] and that of definite Meniere disease is estimated to be 0.034% to 0.19% [5].

Despite the established diagnostic criteria, the transient nature of vertigo often precludes a definitive diagnosis during clinical assessments owing to the absence of observable oculomotor abnormalities. Eye movement observations during vertigo attacks are extremely useful for differentiating between these diseases [6,7].

Recently, the widespread use of smartphones and tablets has fueled rapid advancements in information and data communication technologies. These developments have enabled the growth of digital medical care and telemedicine [8]. Furthermore, external factors, most notably the COVID-19 pandemic, have accelerated this transformation in recent years [9,10]. Previous studies have attempted to record eye movements during attacks using the recording function of cell phones for diagnosing Meniere disease [11]. Shah et al [12] reported 7 cases of BPPV diagnosed with high specificity and sensitivity using eye movement videos captured with a smartphone. Nonetheless, challenges remain in capturing recordings under low-light conditions using smartphones, which limits the universal applicability of these methods [13]. In addition to smartphone-based recordings, various other technologies have been explored in the field of vestibular medicine, including wearable goggles and home-use devices with guided instructions [14]. We recently devised a simple eye movement–recording system that combines a commercially available mini-infrared camera with 3D-printed goggles to record eye movements at home using smartphones [15]. This system allows patients to record their eye movements during vertigo attacks in the dark and provides recorded information to their doctors from home. These approaches reflect the increasing diversity of remote vestibular assessment technologies. However, its widespread adoption and ease of use were limited because the device had to be distributed to patients.

This study devised an eye movement–recording system that uses an iPhone to record eye movements during a vertigo attack. We report the development of an iPhone app that enables the observation and recording of eye movements and head position, along with eco-friendly specialized goggles made of cardboard, specially designed to hold the iPhone. This system is expected to improve vertigo treatment quality by enabling nystagmus monitoring, irrespective of the location and time.

## Methods

### The ICapNYS App That Captures Eye Movements and Head Position

**Figure 1.**
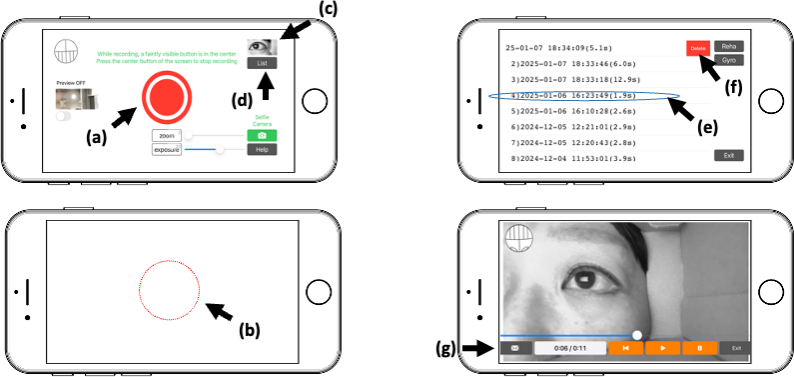
Instructions on how to use the “iCapNYS” application. (a) Tap the red circle button in the center of the screen to start recording. (b) Tap the center of the screen again to end the recording. (c) Tap a thumbnail to check the recorded video. (d) Display the video list. (e) Select and playback. (f) Swipe left to delete. (g) Send the video.

We developed the iCapNYS app to record eye movements and head positions using the iPhone’s front camera and built-in gyro sensor. The app allows for adjustment of focus, exposure, and zoom. The app is available for download from the Apple App Store under the title “iCapNYS.” Since launching the app, the recorded eye movements can be checked with only 3 taps: first, after launching the app, the red circular button at the center of the screen should be tapped to start recording immediately (Figures1A  2A). Next, the center of the screen is to be tapped again to end the recording (Figures1B  2A). Third, the last recorded video can be played back by tapping the thumbnail in the upper-right corner (Figures1C  2A).

**Figure 2.**
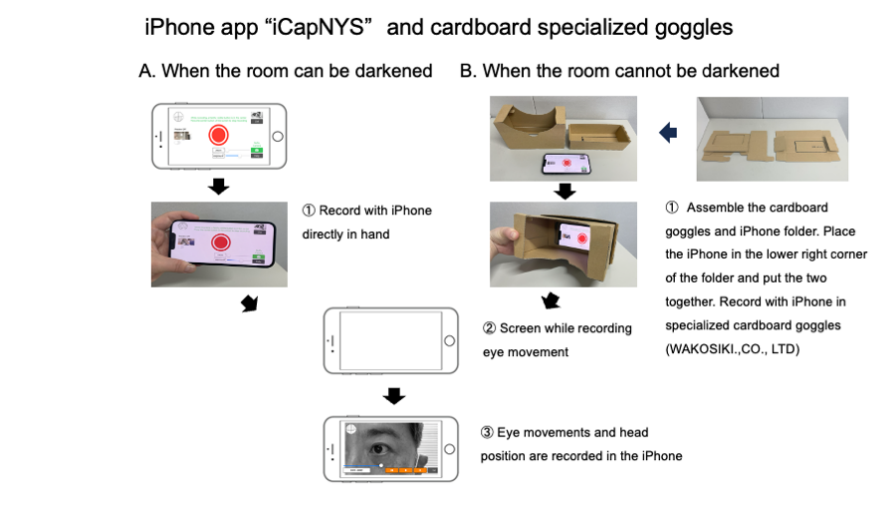
Eye movement video recording during vertigo attacks. (A) If one wants to record easily or can darken the room. (B) If one wants to record more reliably or if the room is brightly lit. The cardboard goggles and iPhone holder must be assembled. The iPhone must be placed in the lower-right corner of the holder and then the two parts must be attached together.

The list of videos can be displayed by tapping the button below (Figure 1D). Next, an item can be tapped to play it (Figure 1E), or the video may be deleted by swiping left (Figure 1F). Additionally, videos are saved in “iCapNYS” albums and can be managed in the Photos app. The recorded videos could be sent to medical professionals using the mail button in the lower-left corner of the video playback screen (Figure 1G).

### “Cardboard Goggles” for iPhone

Specialized goggles made of recyclable cardboard (Wako Shiki Co, Ltd) were used to maintain an optimal positional relationship between the eyeball and the iPhone, ensuring more reliable recording of eye movements. Wearing these specialized goggles prevents fixation suppression caused by the surrounding field of vision. The cardboard goggles consist of 2 parts: a holder for the iPhone and the goggles themselves. These can be easily assembled by the user by folding them along the creases. The iPhone running the app was placed in the lower-right corner of the holder, and the goggles were placed on top for recording. The goggles hold the iPhone in the proper position, enabling more accurate recording of head position changes than direct handheld recording, and allowing for recording eye movements in bright light (Figure 2B). The assembly time was approximately 1 minute (Video 1 in Multimedia Appendix 1). Patients can record their eye movements during head position changes by following the automatic voice guidance by switching to the “automatic 90-seconds” mode with video commentary from the “selfie camera” in the lower-right corner of the iCapNYS app. The voice prompts the user to record eye movements for 15 seconds in the sitting position, followed by supine, right-side supine, left-side supine, and sitting positions, with eye movements recorded for 15 seconds in each position. The system supports audio in Japanese and English.

### Ethical Considerations

This study was conducted with approval from the Medical Research Ethics Committee of Mejiro University (approval number 24 medicine-007). The patient was fully informed of the purpose of the study, its contents, and the handling of the findings, and written informed consent was obtained. All data were treated confidentially and reported anonymously. This study was conducted in accordance with the tenets of the Declaration of Helsinki. Consent to publish their image has been obtained from the individuals pictured in the figures and multimedia appendices. The participant received a JPY 2000 (approximately US $15) gift voucher as compensation.

## Results

To assess the system’s ability to detect pathological nystagmus, we compared nystagmus recordings obtained under infrared video Frenzel glasses with those captured using the iPhone app iCapNYS housed in specialized cardboard goggles (iCapNYS with goggles). The patient was an 82-year-old woman who presented with a vertigo attack attributed to left-sided Meniere disease.

**Figure 3.**
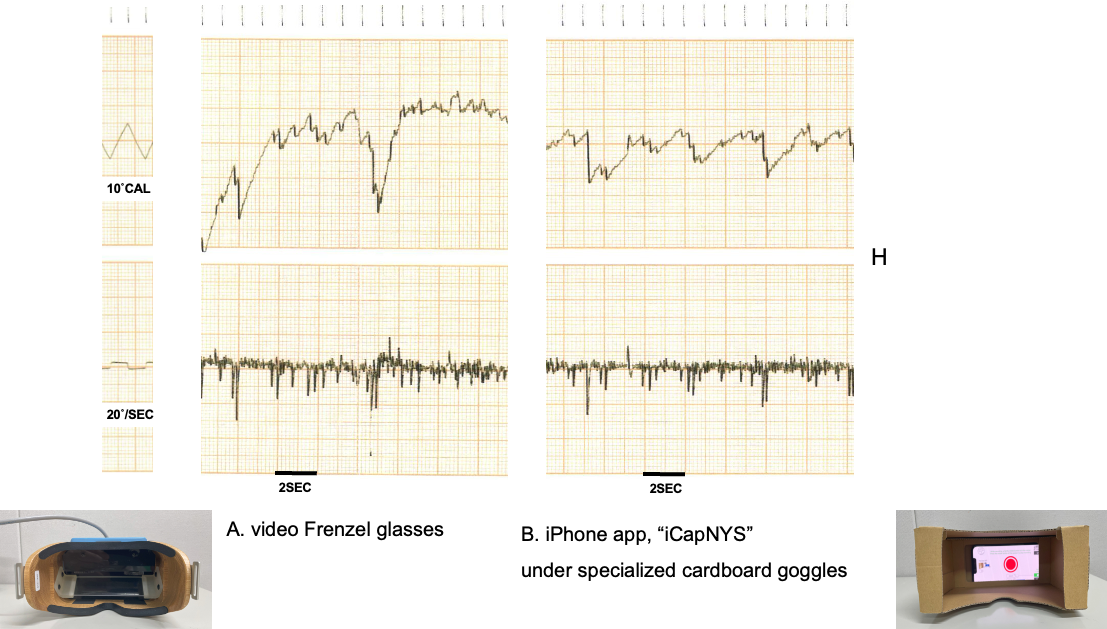
Spontaneous nystagmus in an 82-year-old woman with vertigo. (A) Infrared CCD (charge-coupled device) camera, (B) the “iCapNYS” iPhone app in which nystagmus was simultaneously recorded with electronystagmography in the supine position. Upper panel: eye position. Lower panel: eye velocity waveform.

Initially, no pathological nystagmus was observed during bedside examination under direct visual inspection with the naked eye. However, when the same patient was evaluated using either the infrared video Frenzel glasses or iCapNYS with goggles, fine pathological horizontal nystagmus was clearly visualized. Video recordings were performed in the supine position for approximately 30 seconds using both iCapNYS with goggles and the infrared video Frenzel glasses. Each video was recorded independently (Video 2: infrared video Frenzel glasses in Multimedia Appendix 2 and Video 3: iCapNYS with goggles in Multimedia Appendix 3). With the patient’s cooperation, the pathological nystagmus obtained with both devices was presented as an electronystagmography (ENG) waveform (Figure 3). Recordings obtained from infrared video Frenzel goggles show a distinct leftward horizontal nystagmus with a slow-phase velocity of approximately 12.6°/second (Figure 3A). Comparable waveforms were captured using the “iCapNYS” app under specialized cardboard goggles, with a similar slow-phase velocity of 12.0°/second (Figure 3B). The average number of nystagmus beats during the 30-second recording was 23 for the infrared video Frenzel glasses and 22 for iCapNYS with goggles. These findings suggest that the iCapNYS system can reliably detect subtle peripheral nystagmus and demonstrates performance comparable to that of standard infrared video systems. The specialized goggles with a white screen provided sufficient visual isolation and contributed to the successful detection of nystagmus.

## Discussion

### Principal Findings

Episodic vertigo attacks can occur at any time and in any location. However, dizziness and abnormal results are not always present in patients with vertigo, and several cases exist that are difficult to differentiate or do not result in a definitive diagnosis. In such cases of episodic attacks, it is necessary to differentiate among Meniere disease, vestibular migraine, and other conditions. Therefore, it is extremely useful for patients to record their eye movements during vertigo attacks using this system and provide this information to their physicians for diagnosis and differentiation.

One approach to capturing eye movements during a vertigo attack is to use video recordings from a cell phone or smartphone. For instance, Kıroğlu and Dağkıran [11] showed that recordings made with a cell phone can be used to diagnose Meniere disease. Similarly, Young et al [14] showed that patients could record nystagmus during vertigo episodes using a device attached to an audio or video recorder that incorporated 2 infrared lights within lightweight swimming goggles. They observed that if the head position was simultaneously recorded, distinguishing between normal vestibulo-ocular reflexes and the pathological nystagmus seen in BPPV would be possible, thereby increasing diagnostic accuracy. Our system addresses these difficulties by leveraging a geolocation sensor already integrated into an iPhone, eliminating the requirement for patients to manually record their head positions.

**Figure 4.**
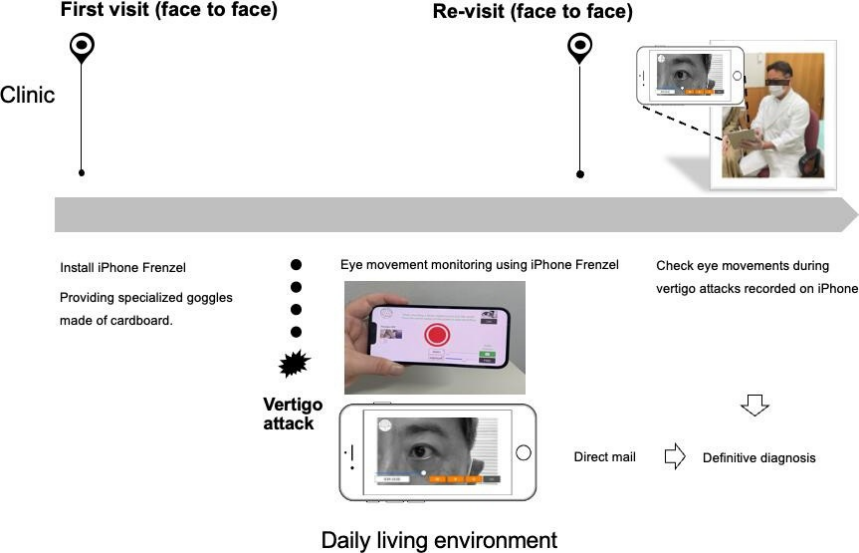
iCapNYS used in episodic vertigo of unknown cause.

In the diagnosis of vertigo, confirming spontaneous nystagmus under nonattentive conditions is especially critical [16]. iCapNYS was specifically designed to reduce gaze-induced nystagmus suppression, enabling patients to record spontaneous eye movements in a nonfixating state during a vertigo attack. iCapNYS does not require being in a dark room and can record even when directly held in front of the patient’s eyes. Moreover, recording stable eye movements is possible using specialized cardboard goggles. Recent studies have underscored the potential of deep learning systems as well as other smartphone-based diagnostic tools for detecting nystagmus [17,18]. Shah et al [12] showed that remote specialists can use smartphone-recorded eye movements to diagnose BPPV. Our system is designed to be operated as easily and accurately as possible by patients themselves during a vertigo attack. Patients can perform practice recordings to familiarize themselves with the procedure, enabling them to record their eye movements during vertigo attacks regardless of location or time. Additionally, the ability to monitor nystagmus enhances the quality of medical care and the accuracy of diagnosis. Although ENG and infrared video Frenzel glasses are typically used by physicians and medical personnel in clinical settings, the proposed device is designed for patient use during actual episodes of vertigo. If a medical system were established in which smartphone-captured eye movements could be emailed to health care providers, appropriate measurements could be taken based on the diagnosis. For example, if a diagnosis of BPPV is confirmed, the patient could be instructed to adopt a head position that does not trigger vertigo and be reassured not to worry. In the future, it may be possible to advise self-treatment through home-based maneuvers in a digital consultation. The introduction of this technology into telemedicine is expected to help build a sense of security and trust between patients and physicians (Figure 4).

### Limitations

Despite these promising developments, the proposed system has certain limitations. The present assessment was based on a single case study, which may not completely represent a broader patient population. Additionally, variations in smartphone hardware and ambient lighting conditions can influence the recording quality, and relying on patients to operate the system during an acute vertigo episode may be challenging, especially for individuals with limited technological proficiency.

The iCapNYS iPhone app, housed in specialized cardboard goggles, has the potential to record subtle peripheral nystagmus in patients with vertigo. However, the lack of infrared capabilities in most smartphones limits their use in complete darkness. Although the cardboard housing is designed to block out ambient light, some leakage may still occur.

### Conclusions

Herein, we developed an innovative eye movement–recording system that uses a patient’s iPhone during a vertigo attack. The system comprises an “iCapNYS” app that captures eye movements and head positions, along with a pair of specialized cardboard goggles specifically designed for the iPhone. We recorded the nystagmus of a patient during a vertigo attack and compared the results with those obtained using conventional infrared video Frenzel glasses to examine the system’s effectiveness. Our findings indicate that the nystagmus frequency and slow-phase velocity recorded using the iCapNYS system were comparable to those captured using traditional methods, with similar visual characteristics observed in both sets of recordings. This system is highly beneficial for treating vertigo, as it enables patients to easily record their eye movements and share data with their physicians at any time and from any location. We anticipate that the widespread adoption of this nystagmus-monitoring system, particularly when integrated into telemedicine, will increase vertigo treatment quality and ultimately foster greater patient confidence and trust.

## Supplementary material

10.2196/73811 Multimedia Appendix 1 Video 1.

10.2196/73811 Multimedia Appendix 2 Video 2.

10.2196/73811 Multimedia Appendix 3 Video 3.

## Abbreviations

BPPV: benign paroxysmal positional vertigo
CCD: charge-coupled device
ENG: electronystagmography
